# Patterning of the Autotrophic, Mixotrophic, and Heterotrophic Proteomes of Oxygen-Evolving Cyanobacterium *Synechocystis* sp. PCC 6803

**DOI:** 10.3389/fmicb.2022.891895

**Published:** 2022-05-25

**Authors:** Dorota Muth-Pawlak, Sanna Kreula, Peter J. Gollan, Tuomas Huokko, Yagut Allahverdiyeva, Eva-Mari Aro

**Affiliations:** Laboratory of Molecular Plant Biology, Department of Life Technologies, University of Turku, Turku, Finland

**Keywords:** photoautotrophy, heterotrophy, photomixotrophy, photosynthesis, carbon metabolism, environmental acclimation, proteomics

## Abstract

Proteomes of an oxygenic photosynthetic cyanobacterium, *Synechocystis* sp. PCC 6803, were analyzed under photoautotrophic (low and high CO_2_, assigned as ATLC and ATHC), photomixotrophic (MT), and light-activated heterotrophic (LAH) conditions. Allocation of proteome mass fraction to seven sub-proteomes and differential expression of individual proteins were analyzed, paying particular attention to photosynthesis and carbon metabolism–centered sub-proteomes affected by the quality and quantity of the carbon source and light regime upon growth. A distinct common feature of the ATHC, MT, and LAH cultures was low abundance of inducible carbon-concentrating mechanisms and photorespiration-related enzymes, independent of the inorganic or organic carbon source. On the other hand, these cells accumulated a respiratory NAD(P)H dehydrogenase I (NDH-1_1_) complex in the thylakoid membrane (TM). Additionally, in glucose-supplemented cultures, a distinct NDH-2 protein, NdbA, accumulated in the TM, while the plasma membrane-localized NdbC and terminal oxidase decreased in abundance in comparison to both AT conditions. Photosynthetic complexes were uniquely depleted under the LAH condition but accumulated under the ATHC condition. The MT proteome displayed several heterotrophic features typical of the LAH proteome, particularly including the high abundance of ribosome as well as amino acid and protein biosynthesis machinery-related components. It is also noteworthy that the two equally light-exposed ATHC and MT cultures allocated similar mass fractions of the total proteome to the seven distinct sub-proteomes. Unique trophic condition-specific expression patterns were likewise observed among individual proteins, including the accumulation of phosphate transporters and polyphosphate polymers storing energy surplus in highly energetic bonds under the MT condition and accumulation under the LAH condition of an enzyme catalyzing cyanophycin biosynthesis. It is concluded that the rigor of cell growth in the MT condition results, to a great extent, by combining photosynthetic activity with high intracellular inorganic carbon conditions created upon glucose breakdown and release of CO_2_, besides the direct utilization of glucose-derived carbon skeletons for growth. This combination provides the MT cultures with excellent conditions for growth that often exceeds that of mere ATHC.

## Introduction

Photosynthetic organisms use light as a source of energy for CO_2_ assimilation, thereby storing light energy into chemical bonds of organic molecules. Nevertheless, several photosynthetic organisms, particularly those from aquatic environments, also possess a capacity for heterotrophic growth or for combining the photoautotrophic (hereafter autotrophic) and heterotrophic traits in a metabolic strategy known as photomixotrophy (hereafter mixotrophy). This capacity can also be used as an asset for fast acclimation of aquatic photosynthetic organisms to changes in environmental conditions, including light intensity and temperature conditions, but particularly to the availability of CO_2_ and other forms of carbon that drastically modulate cell photosynthetic efficiency and growth potential. Metabolic changes that allow for flexibility in the use of carbon from the environment, thereby driving mixotrophy and/or autotrophy strategies, are still poorly understood. Despite several metabolomes (Knoop et al., 2013; Yoshikawa et al., 2013; Nakajima et al., 2014; You et al., 2014; Wan et al., 2017), proteomes (Plohnke et al., 2015; Fang et al., 2017; Toyoshima et al., 2020), and transcriptomes (Yoshikawa et al., 2013; You et al., 2015) studies with model photosynthetic microorganisms, like the prokaryotic cyanobacterium *Synechocystis* sp. PCC 6803 (hereafter *Synechocystis*) investigated here, the choice and regulation of the bioenergetic and metabolic pathways prevailing under a particular trophic mode have remained poorly understood, mainly because of the complexity of experimental questions and designs involved in the deeper elucidation of different trophic strategies. Cyanobacteria are the only prokaryotes capable of oxygenic photosynthesis, and, although the comparison of their metabolism with that of the best investigated heterotrophic bacterium *Escherichia coli* has revealed distinct differences, it has also highlighted numerous knowledge gaps concerning the functionality of cyanobacterial metabolism (Mills et al., 2020).

During the past decade, cyanobacteria have attracted special attention as potential future photosynthetic cell factories, which, *via* synthetic biology approaches, will be engineered to efficiently convert solar energy into useful chemicals and fuels (Gangl et al., 2015; Aro, 2016; Lea-Smith and Howe, 2017; Lindblad et al., 2019; Santos-Merino et al., 2019; Liu et al., 2022). The capability of cyanobacteria to adopt different trophic strategies is used as an asset in enhancing productivity in photosynthetic cell factories and different co-culture designs, for example, with yeast (Song et al., 2014; Hays et al., 2017; Ai et al., 2021). Nevertheless, the prevalence and modulations of different metabolic and bioenergetic routes under different trophic conditions have remained rather undefined. However, this knowledge is extremely relevant for the design and implementation of new biotechnology methods when endogenous cell metabolism should be fluently integrated with newly introduced metabolic traits in photosynthetic microorganisms.

Flux of energy for cellular needs is the key difference among different trophic growth modes. Autotrophic traits of harvesting and transduction of light energy in cyanobacteria are confined to the thylakoid membrane (TM), while respiratory pathways are present in both the TM and the plasma membrane (PM) (Schultze et al., 2009; Mullineaux, 2014). The extent to which these pathways are modified in different trophic modes (Plohnke et al., 2015; Huokko et al., 2017, 2019), has not been thoroughly investigated. Similarly, the carbon assimilation and hydrocarbon catabolic pathways as well as many other pathways of central metabolism are expected to be modulated depending on trophic growth condition.

*Synechocystis* glucose-tolerant strain is one of the most extensively investigated cyanobacterial species. In addition to autotrophic (AT) and mixotrophic (MT) growth, *Synechocystis* is capable of light-activated heterotrophic (LAH) growth in darkness, provided that cells are supplemented with a short light period every 24 h (Anderson and McIntosh, 1991). Here, we performed a comparative proteomics study to dissect proteins and corresponding pathways that are important specifically for AT, MT, and LAH strategies in support of *Synechocystis* growth at a similar OD_750_ close to 1. Additionally, the availability of inorganic carbon (C_i_) as CO_2_ at low (0.04%, ATLC) or high (3%, ATHC) levels was investigated under autotrophy. With respect to the general availability of carbon, the ATHC, MT, and LAH trophic conditions were collectively called carbon-rich conditions irrespective of organic or inorganic origin, whereas ATLC represented the natural low-carbon reference condition. LAH, on the contrary, was the only condition with strongly limited availability of light, whereas the other trophic strategies were provided with equivalent irradiance conditions.

## Materials and Methods

### Cultivation Conditions

In this study, we used the glucose-tolerant cyanobacterial strain *Synechocystis* sp. PCC 6803. Pre-experimental cultures were grown in a BG11 medium buffered with 20 mM HEPES-NaOH (pH 7.5) under continuous white light of 50 μmol photons m^−2^ s^−1^ at 30°C, under air enriched with 3% CO_2_ with agitation at 150 rpm for 3 days. Experimental cultures were inoculated at OD_750_ = 0.1 in a 100-ml fresh BG11 medium buffered with 20 mM HEPES-NaOH (pH 7.5) and agitated in 250-ml Erlenmeyer flasks at 150 rpm in AlgaeTron AG130 (PSI Instruments, Czech) cool-white LED growth chambers at 30°C. The cultures were cultivated under four different conditions; (i) autotrophic growth at low (0.04%) CO_2_ (ATLC) and continuous illumination (50 μmol photons m^−2^ s^−1^); (ii) autotrophic growth under high (3%) CO_2_ conditions (ATHC) and continuous illumination (50 μmol photons m^−2^ s^−1^); (iii) mixotrophic growth (MT) in the presence of 10 mM glucose (Glc) at low (0.04%) CO_2_, and continuous illumination (50 μmol photons m^−2^ s^−1^); (iv) light-activated heterotrophic growth (LAH) in the presence of 10 mM Glc at low (0.04%) CO_2_ in the dark, where cells were illuminated (50 μmol photons m^−2^ s^−1^) for only 10 min every 24 h. Optical densities at 750 nm (OD_750_) were measured using a Lambda 25 UV/VIS (PerkinElmer) spectrometer. The cultures were grown under the experimental conditions described above until OD_750_ ≈ 1, taking 1.5 days for the MT and ATHC conditions or 3.5 days for the ATLC and LAH conditions.

To determine the influence of inoculation of experimental cultures with HC-adapted cells on the proteome of ATLC experimental cultures, the following experimental setup was used. Two types of pre-cultures were grown under the ATLC and ATHC conditions, and each of them was further inoculated in a BG11 medium and grown under ATLC conditions (as described above). The cells were collected at OD = 1 at c.a. 1.5 days.

### Mass Spectrometry Data-Dependent Acquisition

For liquid chromatography-tandem mass spectrometry (LC-ESI-MS/MS) analysis, total proteins were isolated and digested as described previously (Huokko et al., 2019).

LC-ESI-MS/MS analyses were performed on a nanoflow HPLC system (Easy-nLC1200, Thermo Fisher Scientific) coupled to a Q Exactive HF mass spectrometer (Thermo Fisher Scientific) equipped with a nano-electrospray ionization source. Samples from three to four individual cultures of each condition (*n* = 3 or 4) were injected into an analytical C18 column (75 μm ×40 cm, ReproSil-Pur 1.9 μm 120 Å C18-AQ; Dr. Maisch HPLC GmbH, Ammerbuch-Entringen, Germany). The mobile phase consisted of water with 0.1% formic acid (solvent A) or acetonitrile/water [80:20 (v/v)] with 0.1% formic acid (solvent B). Peptides were separated with a two-step, 110 min gradient from 5 to 26% solvent B for over 70 min, followed by 26–49% B increase for over 30 min.

MS data were acquired automatically using the Thermo Xcalibur 3.1 software (Thermo Fisher Scientific). A data-dependent acquisition (DDA) method consisted of an Orbitrap MS survey scan of mass range 300–1,800 m/z followed by HCD fragmentation for the 12 most intense peptide ions. Spectra were registered with a resolution of 120,000 and 15,000 (at m/z 200) for full scan and for fragment ions, respectively, and normalized collision energy of 27%. Automatic gain control (AGC) was set to a maximum fill time of 100 ms and 250 ms to obtain a maximum number of 3e6 and 1e5 ions for MS and MS/MS scans, respectively.

### Data Analysis

Proteins were identified and annotated according to the *Synechocystis* database retrieved from Cyanobase (Kaneko et al., 1996) (3,672 entries, 23.10.2012) using the Proteome Discoverer 2.2 software (Thermo Fisher Scientific) connected to an in-house server running the Mascot 2.6.1 (Perkins et al., 1999) algorithm (Matrix Science). Precursor value was restricted to monoisotopic mass tolerance of ±4 ppm and fragment ion mass tolerance of ±0.02 Da. Two missed cleavages were allowed, and decoy searches were performed. For validation of the identified spectra, we used a Percolator algorithm (Käll et al., 2007) with a relaxed false discovery rate (FDR) of 0.05. The original data and protein identification files are deposited in the PRIDE Archive database (Vizcaíno et al., 2016) (PXD030630). A quantitative analysis was conducted in the Progenesis (Waters) software with global normalization and using relative quantification of proteins with at least two peptides with no conflicts per protein. The statistical test ANOVA was used (with experimental setup called in Progenesis software as between subject design), assuming that the tested conditions are independent. In the analysis of different proteomic responses of *Synechocystis* upon acclimation to various trophic conditions, the practical threshold of fold change (FC) was set to 1.5 (log_2_1.5 =0.58) for upregulated proteins and −1.5 [–log_2_(1.5) = −0.58] for less abundant proteins. Proteins quantified in at least three replicates and at least two conditions were used for data interpretation. Data with missing points or unreliable peptides were filtered out before the quantification process.

Obtained values of protein abundance were also used to calculate the distribution of proteome mass fraction to functional categories (Jahn et al., 2018) under the four conditions: ATLC, ATHC, MT, and LAH. Proteins were grouped according to an annotation used previously (Jahn et al., 2018) (Supplementary Table 1).

## Results

### Growth of *Synechocystis* Cultures Under Different Trophic Conditions

**Figure 1:**
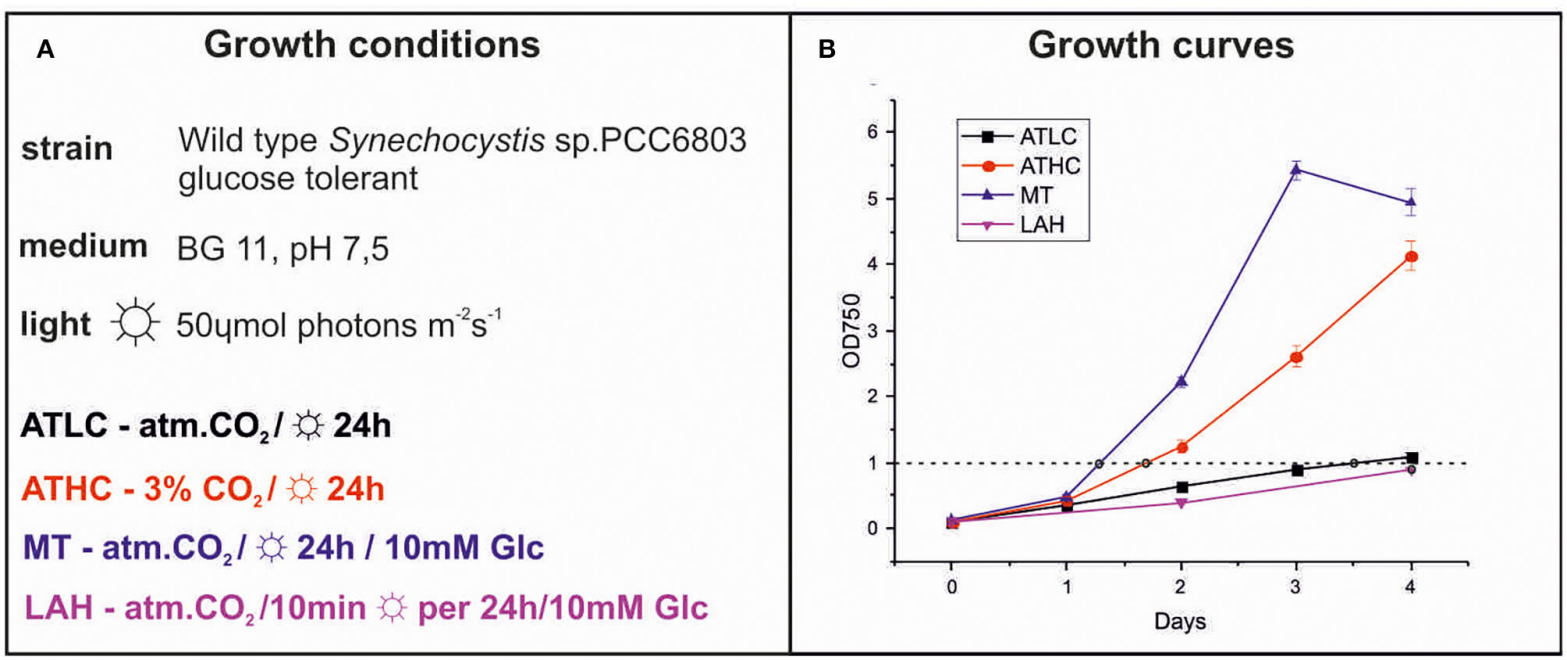
Different growth strategies in Synechocystis cells. Growth conditions **(A)** and growth dynamics **(B)** of Synechocystis cells under photoautotrophic at low CO_2_ (ATLC; black) and high CO_2_ (ATHC; red), photomixotrophic (MT; blue) and light activated growth (LAH; magenta) conditions. The timing of collection of cells for proteomics, when optical density at 750 nm (OD_750_) 1,0 is marked with black circles. Values are means ± SD; *n* = 3 biologically independent experiments.

Four different growth strategies were applied to *Synechocystis* to investigate the proteome pattern of cultures upon reaching the optical density at 750 nm (OD_750_) of ca. 1 (Figure 1). *Synechocystis* cells were grown autotrophically under air-level (0.04%) (ATLC) or enriched 3% CO_2_ (ATHC), or mixotrophically with 10 mM Glc (MT) or under light-activated heterotrophy (LAH) with 10 mM Glc (Figure 1A); the latter two were also under air-level CO_2_. The ATHC and MT cultures exhibited rapid growth, which was attributed to excess of available inorganic and organic carbon, respectively (Figure 1B). On the contrary, the slower growth of *Synechocystis* under the ATLC and LAH conditions was due to limited carbon and light for photosynthesis, respectively (Figure 1). Cells were harvested for proteome analysis when OD_750_ ≈ 1 had been reached after about ≈1.5 days of growth under the ATHC and MT conditions and ca. 3.5 days under ATLC and LAH conditions (Figure 1B). Notably, the pre-cultures were grown under HC while the growth of experimental cultures took place at LC in the case of ATLC, MT, and LAH. This prompted us to test whether the pre-cultures grown under LC or HC influenced the proteome of ATLC to be analyzed after the OD_750_ ≈1.0 had been reached. Quantitative analysis of *Synechocystis* autotrophic proteomes from cells acclimated to the LC condition and from cells acclimated to HC and shifted to the LC condition revealed (at OD_750_ = 1) only minor differences (Supplementary Table 2). Seventy-nine proteins were differentially expressed and included glutamate-ammonia ligase (GlnN) and glutamine synthetase inactivating factors GifA and GifB as well as potassium and sulfate transporters. Notably, no differences were recorded in carbon metabolism or photosynthetic/respiratory proteins. Thus, we chose to use HC-acclimated cells for inoculations in analyses of different trophic proteomes of *Synechocystis*, keeping in mind the minor effects of the pre-culturing condition.

### General View of *Synechocystis* Proteome Under the Four Growth Strategies

**Figure 2:**
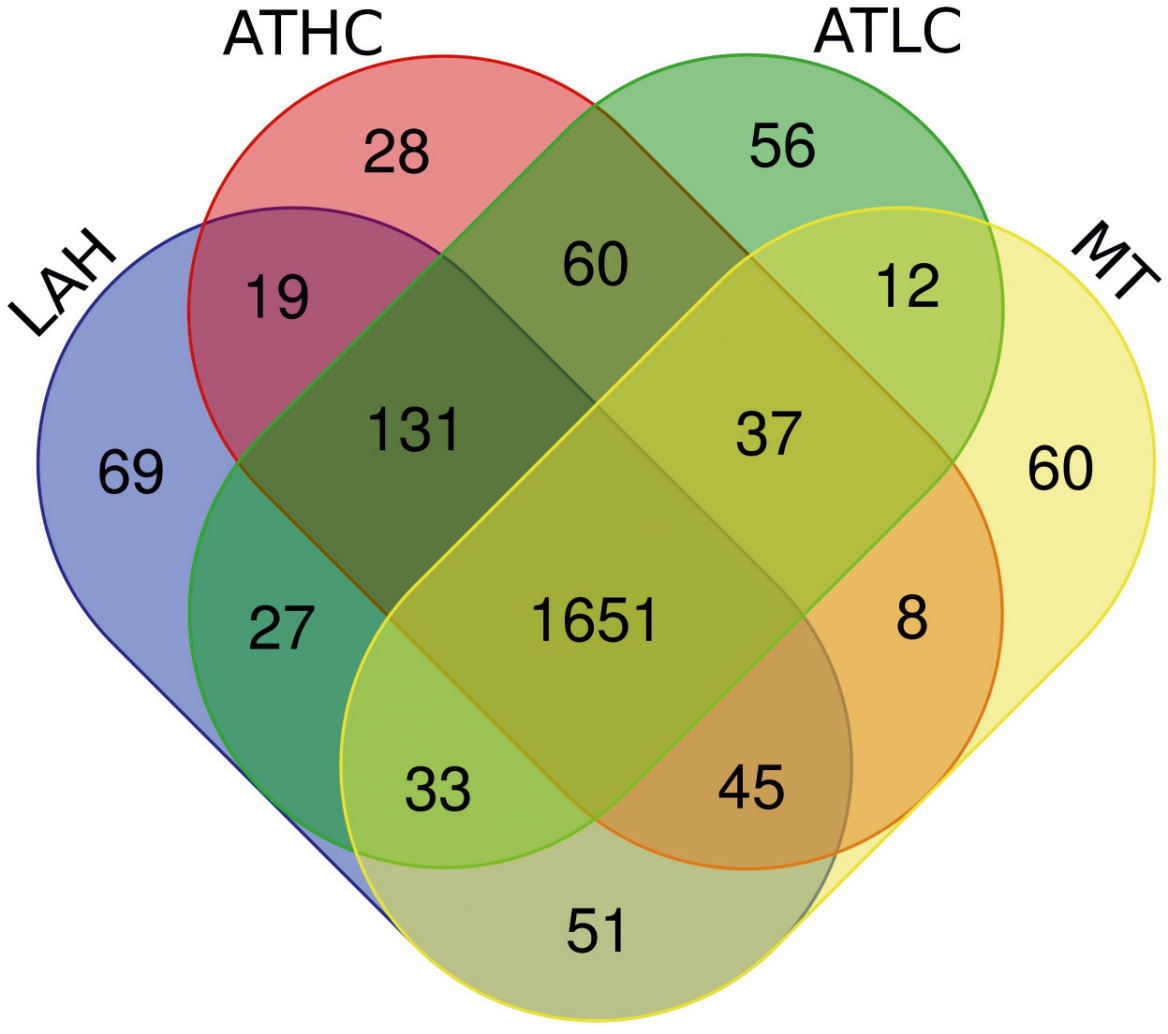
Venn diagram showing the number of proteins identified under the four growth conditions, ATLC, ATHC, MT, and LAH.

Overall, 2,287 proteins (62% of *Synechocystis* theoretical proteome) were identified with at least two peptides present in at least two conditions under study (Supplementary Tables 3–7). Of these, 1,651 proteins (45% of *Synechocystis* proteome) were identified in all the growth modes, as shown in the Venn diagram summarizing all proteins identified in this study (Figure 2). A total of 2,145 proteins were quantified by label-free DDA mass spectrometry represented by at least 2 peptides (ANOVA *P* < 0.05) (Supplementary Table 1).

### Proteome Mass Fraction Analysis Revealed Growth Condition-Dependent Protein Accumulation in Different Cellular Functionalities

As the first approach, we analyzed the distribution of protein mass into different functional categories (main and subcategories as classified by Jahn et al., 2018) and calculated the fraction of the total proteome mass allocated to the seven main functional categories for every growth condition (ATLC, ATHC, MT, and LAH) (Table 1).

**Table 1:** Proteome classification categories used for mass fraction analyses.

**Proteins classification categories**		
PSET	CYTB6F	Cytochrome b_6_f
	SEC	Soluble electron carriers
	NADH	Respiartoy NDH-1 and 2
	RTO	Respiratory terminal oxidases
	HYDR	Hydrogenase
	ATPS	ATP synthase
	PSI	Photosystem I
	PSII	Photosystem II
LHC	PBS	Phycobilisome
	BSC	Biosynthesis of cofactors (chlorophyll)
CBM	TBP	Transport and binding proteins
	CCM	Carbon concentrating mechanism
	AAB	Aminocids biosynthesis
	CIM	Central intermediary metabolism
	TS	Translation
	EM	Energy metabolism
GLM	GLC	Glycolysis
	OPP	Pentose phosphate pathway
	SUGMET	Sugars
LPB	CE	Cell envelope
	BSC	Biosynthesis of cofactors (Lipoate)
	FA	Fatty acid
MAI	BSC	Biosynthesis of cofactors (others)
	PPN	Purines, pyrimidines, nucleosides and nucleotides
	CELPROC	Cellular processes
	TRANSL	Translation
	CIM	Central intermediary metabolism
	TRANSCR	Transcription
	OTHER	Other categories
	HYP	Hypothetical
	RF	Regulatory functions
	DNARP	DNA replication, restriction, modification, Recombination, and repair
	UNK	Unknown
	WDPROT	WD repeat proteins
RIB	CELPROC	Cellular processes
	TRANS	Translation (ribosomal proteins)

*PSET, photosystems and photosynthetic transport; LHC, light-harvesting complex; CBM, carbon metabolism; GLM, glucose uptake and metabolism; LPB, lipid biosynthesis and membrane components; MAI, maintenance and regulation (including proteins of unknown function and hypothetical); RIB, ribosome and protein production*.

**Figure 3:**
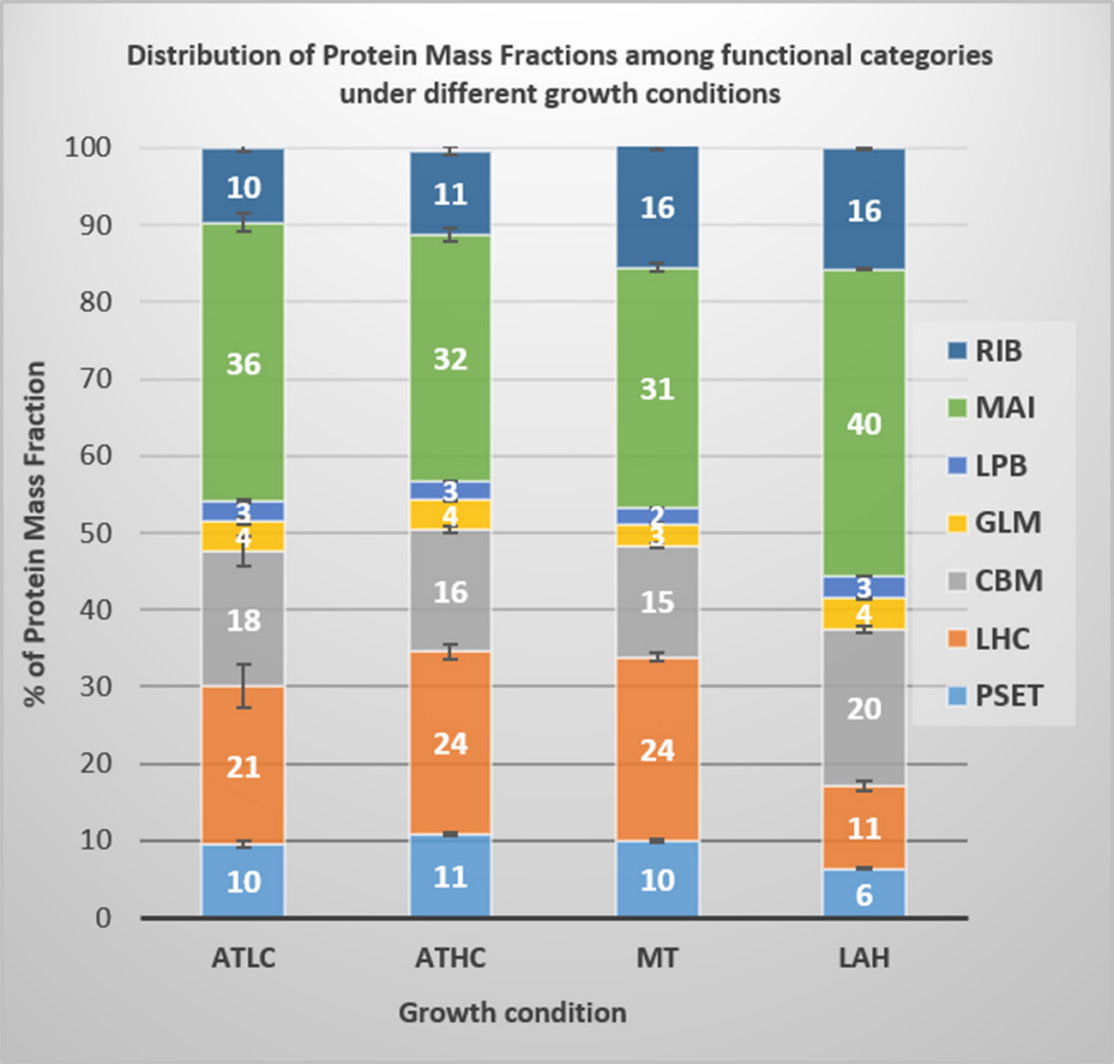
Proteome mass fraction distribution into main categories in *Synechocystis* cells grown under the four different growth conditions, ATLC, ATHC, MT, and LAH. Functional categories: PSET, photosystems and photosynthetic transport; LHC, light-harvesting complex; CBM, carbon metabolism; GLM, glucose uptake and metabolism; LPB, lipid biosynthesis and membrane components; MAI, maintenance and regulation (including proteins of unknown function and hypothetical); RIB, ribosome and protein production. Three to four biological replicates per condition were used to calculate proteome mass fractions and corresponding standard error.

This analysis was conducted to reveal functionalities that either increased or decreased in the *Synechocystis* proteome under the different growth modes and thus, gave insights into the allocation of cell's energetic and material resources to the particular functional category under each cultivation strategy. The measurement of mass fraction is based on the assumption that the overall amount of proteome mass in *Synechocystis* cells does not fluctuate under the four different growth conditions when a OD_750_ of 1 had been reached (Zheng and O'Shea, 2017). Figure 3 depicts the percentage of proteome mass fraction distribution in the main functional categories shown in Table 1.

As expected, the allocation of proteome mass fractions in LAH distinctively differed from those under the ATLC, MT, and ATHC conditions. In particular, the proteome mass fractions collectively allocated to photosynthetic electron transfer (PSET) and light-harvesting phycobilisomes (LHC) were reduced to 17% in LAH in comparison to the 31–35% in the remaining conditions (Figure 3). Conversely, LAH uniquely accumulated proteome fractions in the maintenance (MAI) category, comprising many proteins of unknown function, as well as in the carbon metabolism (CBM) category (Supplementary Table 1). Relatively high proteome mass fraction of the ribosome and protein production (RIB) category was a common feature for both MT and LAH (Figure 3). The ATHC cultures, despite rapid cell proliferation close to that of MT (Figure 1B), demonstrated a lower mass fraction of the RIB category (Figure 3). The two autotrophic conditions, ATLC and ATHC, differed by a decrease in LHC proteome fraction and accumulation of MAI and CBM proteome fractions in the slowly growing ATLC cultures, in comparison to the faster-growing ATHC cultures.

**Figure 4:**
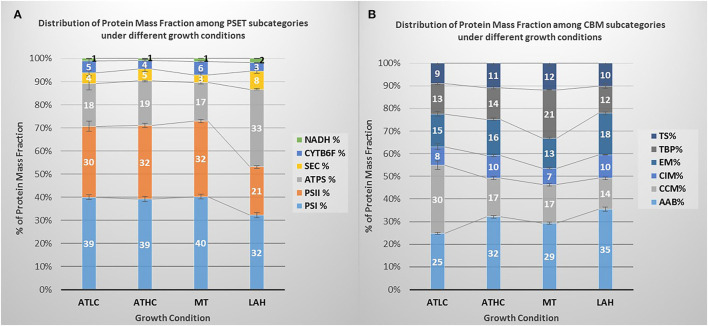
Distribution of protein mass fraction in categories of **(A)** photosystems and photosynthetic transport (PET) and **(B)** relevant subcategories of carbon metabolism (CBM) under the different growth conditions: ATLC, ATHC, MT, and LAH. In **(A)**, PSI, photosystem I; PSII, photosystem II; ATPS, ATP synthase; SEC, soluble electron carriers, Cyt*b*_6_*f*-cytochrome *b*_6_*f* , NADPH-dehydrogenase complex (NDH-1_1_ and NDH-2); in **(B)**, AAB, amino acid biosynthesis; CCM, carbon-concentrating mechanism; CIM, central intermediary metabolism; EM, energy metabolism; TBP, transport and binding proteins; TS, translation. Three to four biological replicates per condition were used to calculate proteome mass fractions and corresponding standard error.

The two major functional categories, PSET and CBM, were selected for deeper analyses of proteome mass fraction accumulation into their various protein subcategories shown in Table 1. Differential proteome mass accumulation in the subcategories of PSET and CBM is presented in Figures 4A,B, respectively. In the PSET category, both photosystem II (PSII) and photosystem I (PSI) collectively accumulated to a substantially lower amount in LAH (49%) than in the ATLC (60%), ATHC (68%), or MT (69%) condition (Figure 4A). At the same time, ATP synthase fraction (ATPS), as well as the respiratory complexes NDH-1_1−2_ and NDH-2 proteins, increased under LAH as compared to the other conditions. The CBM category (Figure 4B) demonstrated relatively high proteome mass fractions allocated to the amino acid biosynthesis (AAB) in ATHC, MT, and LAH in comparison to ATLC. On the contrary, the protein mass fraction of the carbon concentration mechanism (CCM) subcategory was substantially larger in ATLC (30%) compared to the three carbon-rich growth conditions (14–17%). The allocation of protein mass into the transport and binding protein (TBP) subcategory was higher in MT (21%) than in the other growth conditions (12–14%).

## Trophic Mode-Dependent Differential Protein Expression

After focusing on functional categories, we analyzed the differential expression of individual proteins in *Synechocystis* in response to growth under ATHC, MT, and LAH using the protein abundance in *Synechocystis* grown autotrophically at low CO_2_ (ATLC) as a reference. Results on differentially expressed (DE) proteins are presented below in Tables 2–6, grouping the proteins primarily according to the main functional categories (see Table 1).

**Table 2:** Differentially expressed proteins from the carbon metabolism (CBM) category.

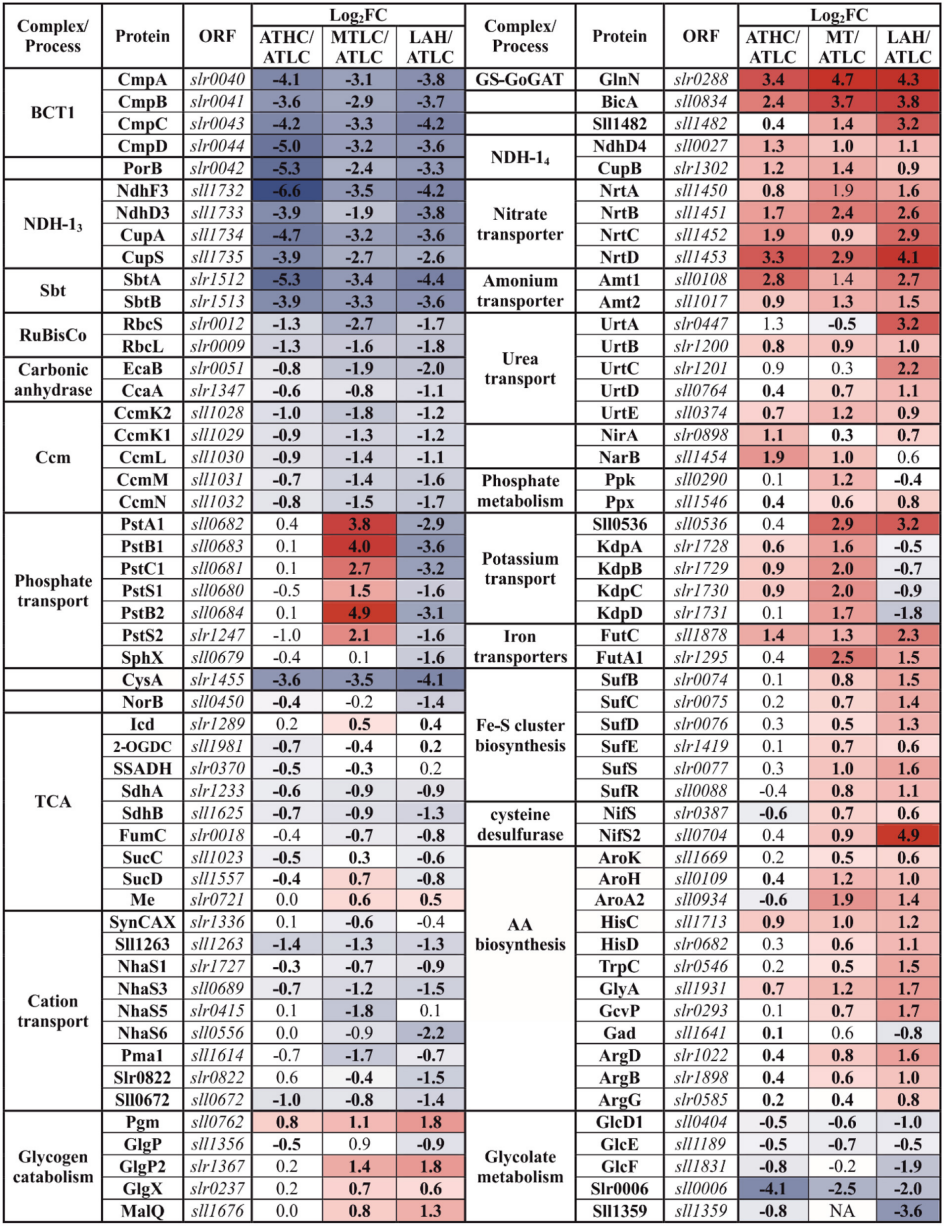

*The gradient from blue to red was applied to highlight the proteins below or above practical fold change threshold (−0.58 > log_2_FC >0.58), with lowest to highest abundance, respectively, in relation to the ATLC growth condition. Statistically significant results (p <0.05) are highlighted with bold font*.

### Differential Expression of Carbon Metabolism-Related Proteins in Response to the Trophic Strategy

Proteins of the constitutively expressed C_i_ uptake systems (BicA and NDH-1_4_ complex) were particularly abundant under all the carbon-rich conditions (ATHC, MT, and LAH) with respect to the ATLC reference condition (Table 2). Conversely, inducible components of inorganic CCM, such as NDH-1_3_ and bicarbonate transporters BCT1 and SbtA/SbtB, had very low abundance under ATHC, MT, and LAH. Intriguingly, the porin protein PorB, a product of the *cmp* operon that encodes the BCT1 transporter, showed the lowest differential expression in ATHC. Ribulose bisphosphate carboxylase (RuBisCO) subunits, RbcS and RbcL, and structural proteins of carboxysome CcmK-N were less abundant under ATHC than under the ATLC conditions and clearly the least abundant under the LAH and MT trophic modes. Similarly, both carbonic anhydrases, the thylakoid-located EcaB (Sun et al., 2019) and CcaA, showed lower abundance under ATHC and even more so under MT and LAH. Importantly, enzymes of glycolate metabolism involved in photorespiration, particularly glyoxylate aminotransferase (Slr0006) (Eisenhut et al., 2008), showed downregulation under all the carbon-rich conditions compared to control ATLC.

Glutamate-ammonia ligase GlnN, which is the primary enzyme in nitrogen assimilation *via* the GS-GOGAT pathway, maintained a high expression level under all the carbon-rich trophic conditions, but showed downregulation under the ATLC condition (Table 2; Supplementary Table 1). Different mechanisms of nitrogen uptake, including the high-affinity nitrate/nitrite transporter NrtA-D, as well as the ammonium permeases Amt1 and Amt2, were also among the most elevated proteins under the ATHC, MT, and LAH conditions in relation to ATLC, while the urea transporter UrtA-E had highest abundance under LAH and moderately upregulated under the MT and ATHC conditions compared to ATLC. The nitrate/nitrite reductases NirA and NarB were upregulated under all the carbon-rich conditions.

Phosphate transport systems, on the other hand, showed the most differential expression under the three carbon-rich conditions in comparison to ATLC (Table 2). The high-velocity, low-affinity Pst1 phosphate transport system (SphX, PstS1, PstC1, PstA1, PstB1, and PstB2) encoded by the *pst1* gene cluster as well as the low-velocity, high-affinity Pst2 system encoded by a homologous *pst2* gene cluster (*slr1247*–*slr1250*) (Pitt et al., 2010) accumulated strongly under the MT growth condition, underwent a significant decrease in abundance under LAH, and remained unaffected by ATHC (Table 2). The phosphate-binding PstS homolog was found with reduced abundance under ATHC and LAH, while the exopolyphosphatase Ppx had high abundance under all the carbon-rich conditions, and polyphosphate kinase Ppk was induced under MT but decreased under LAH, all in comparison to the ATLC trophic mode.

The tricarboxylic acid (TCA) cycle oxidizes acetyl-CoA released from carbohydrates and forms 2-oxoglutarate (2-OG), an intermediate metabolite that functions as a reporter of cellular carbon to nitrogen C/N balance and carbon skeleton for nitrogen assimilation (Muro-Pastor et al., 2001; Fokina et al., 2010; Huergo and Dixon, 2015). Enzymes related to the transformation of 2-OG to succinate *via* succinate-semialdehyde dehydrogenase (SSADH) bypass, including 2-OG decarboxylase (2-OGDC) and SSADH (Xiong et al., 2014), had significantly lower levels under ATHC, while both succinate dehydrogenase subunits ShdA/B and fumarase (FumC) had a low level under all the three carbon-rich conditions in comparison to ATLC (Table 2). At the same time, the amount of succinyl-CoA synthetase SucC/D (of the TCA cycle) was clearly reduced only under LAH. From the GABA shunt of the TCA cycle, only N-acetylornithine aminotransferase (ArgD) had significantly higher abundance under MT and even more under LAH. In addition, an N-acetylglutamate kinase (ArgB) increased in abundance under the LAH condition (at the border of practical threshold under the MT condition). At the same, time the malic enzyme (Me) from the malic shunt of the TCA cycle was slightly upregulated under the MT condition, indicating enhanced flow through the malic shunt (You et al., 2015). Furthermore, multiple enzymes involved in AAB pathways were induced under Glc supplemented conditions MT and LAH (Table 2).

As expected, the LAH and MT conditions enhanced the abundance of enzymes involved in glycogen catabolism, including the glycogen phosphorylase 2 (GlgP2) isoform, phosphoglucomutase (Pgm), 4-alpha-glucanotransferase (MalQ), and glycogen isoamylase (GlgX). At the same time, the other GlgP isoform (encoded by *sll1356*) was downregulated under the ATHC and LAH conditions, in line with a previous report showing the highest activity of GlgP under autotrophic conditions (Fu and Xu, 2006).

Coordinated downregulation of proteins responsible for cation transport, such as Na^+^/H^+^ antiporters (NhaS), Sll1263, PacL, Pma1, and SynCAX, was observed under all the carbon-rich conditions (Table 2). At the same time specifically potassium-transporting P-type ATPase subunits of KdpA-D were strongly induced under MT but strongly decreased under LAH and ATLC. In addition, the KchX potassium channel (Berry et al., 2003) was upregulated in MT and LAH (Table 2).

From other CBM category proteins of high physiological importance, a nitric oxide reductase (NorB), a protein reducing nitrogen oxide (NO) and operating in connection to the electron transport chain in TM (Büsch et al., 2002), was downregulated under the LAH condition (Table 2). The sulfate transporter CysA, in turn, was maintained at a low level under all the carbon-rich conditions. In contrast, iron transporters FutA1 and FutC were induced under all three carbon-rich conditions, which corresponds with upregulation of the *suf* operon (*slr0074-slr0077*) that is important for the biosynthesis of Fe-S clusters. Cysteine desulfurase (NifS2) and an ABC-transporter DevC-homolog, Sll1482, were among the most upregulated proteins in the CBM category under the LAH condition.

### Trophic Strategy-Response of Glucose Uptake and Metabolism (GLM)-Related Proteins

Enzymes of the glycolytic routes, the Embden-Meyerhof-Parnas (EMP) pathway, Entner-Doudoroff (ED) pathway, and oxidative pentose phosphate (OPP) pathway, showed variance in abundance among the three different carbon-rich conditions (Table 3). As for individual GLM category proteins, phosphomannose isomerase (RfbM), which catalyzes the conversion of mannose-6-P to fructose-6-P, was found in higher abundance under the MT and LAH conditions, while under the ATHC condition fructose-bisphosphate aldolase (Fda) was observed at a higher level than under ATLC. Phosphofructokinase (Pfk1) and phosphoglycerate mutase (Pgm) were among the most abundant proteins of the GLM subcategory under all the carbon-rich conditions. On the contrary, two UDP-glucose 4-epimerases (Sll0244 and Slr1067) and hypothetical protein Slr1617, as well as GDP-mannose 4,6-dehydratase (RfbD) were among the most downregulated proteins in comparison to ATLC. The enzyme glucokinase (Glk) that phosphorylates glucose to prevent its diffusion from cells, was downregulated under LAH and MT as compared to ATLC. Common enzymes leading to pyruvate production in glycolysis, such as phosphoglycerate kinase (Pgk) and enolase (Eno), were present at lower abundance in Glc-supplemented growth modes in comparison to the autotrophic ATLC and ATHC conditions.

**Table 3:** Differentially expressed proteins from the glucose uptake and metabolism (GLM) category.

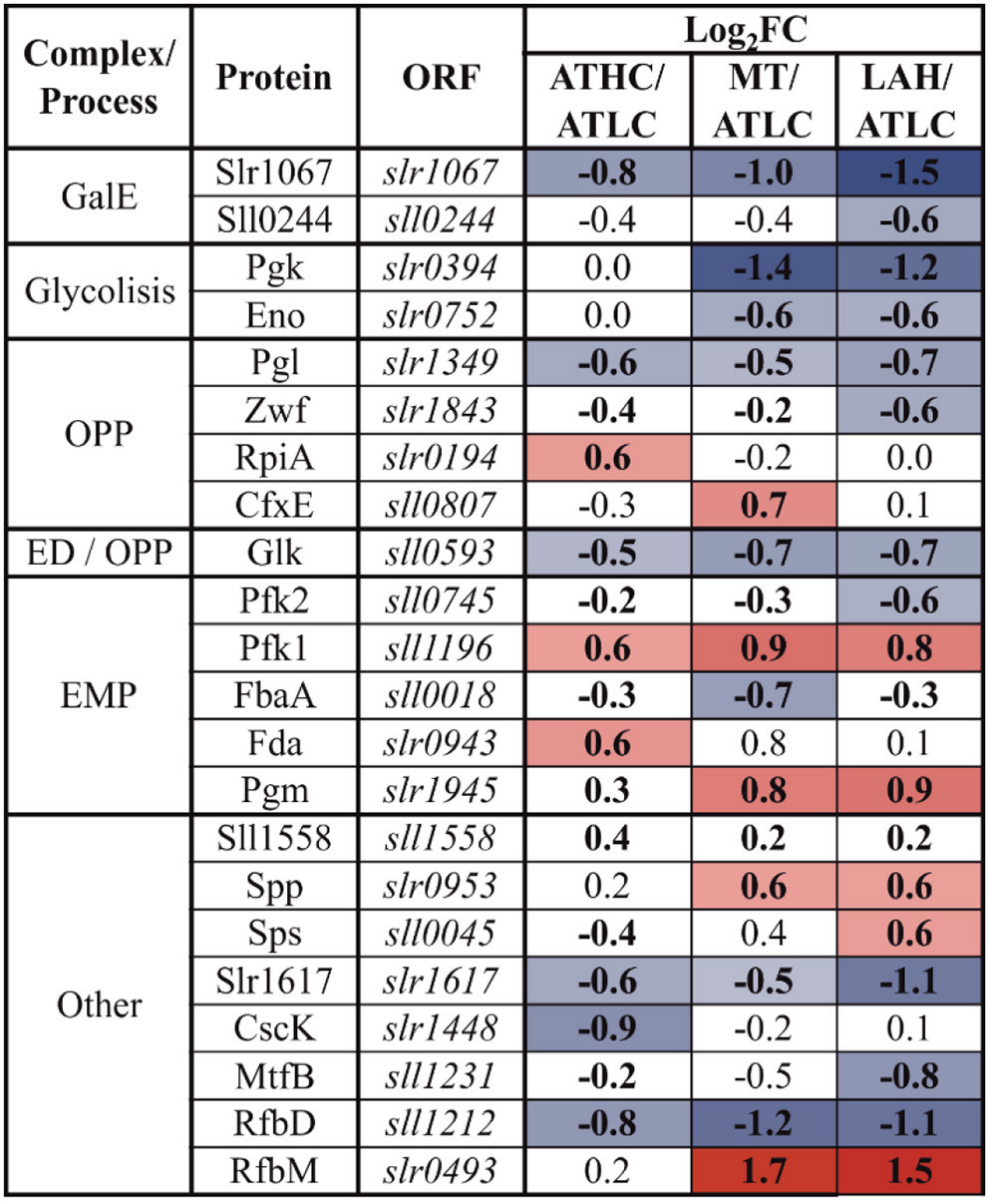

*The gradient from blue to red was applied to highlight the proteins above the practical fold change threshold (−0.5 8> log_2_FC > 0.58), with lowest to highest abundance, respectively, in relation to the ATLC growth condition. Statistically significant results (p <0.05) are highlighted with bold font*.

### Trophic Strategy-Dependent Expression of Photosynthetic, Respiratory, and Tetrapyrrole Biosynthesis Proteins (PSET and LHC)

All protein components of PSII and PSI as well as phycobiliproteins (PBS) demonstrated lower abundance under LAH than under the ATLC condition, with PSII subunits more strongly downregulated than PSI and PBS subunits (Table 4). On the contrary, under the ATHC condition, several components of PSI, PSII, and PBS complexes had higher abundance than under ATLC. Interestingly, PSII-associated proteins encoded by the *Pap* operon (*slr0144-slr0151*) showed higher abundance under all the carbon-rich trophic strategies compared to ATLC and were among the most upregulated proteins in the PSET category (Table 4). Similar behavior was demonstrated by a protein, BtpA, involved in the biogenesis of PSI (Bartsevich and Pakrasi, 1997). In addition, the PSII assembly factor (PSII AF) Pam68 (Rengstl et al., 2013) was elevated under the ATHC condition, while Ycf39 (Knoppová and Komenda, 2019) was downregulated under the LAH and MT conditions (Table 4). Abundances of Psb28-2 and phycocyanin lyase subunits (CpcE/F) were distinctively elevated under the Glc-supplemented MT and LAH conditions compared to ATLC.

**Table 4:** Differential expression of photosynthetic and respiratory proteins from the photosystems and photosynthetic transport (PSET) category.

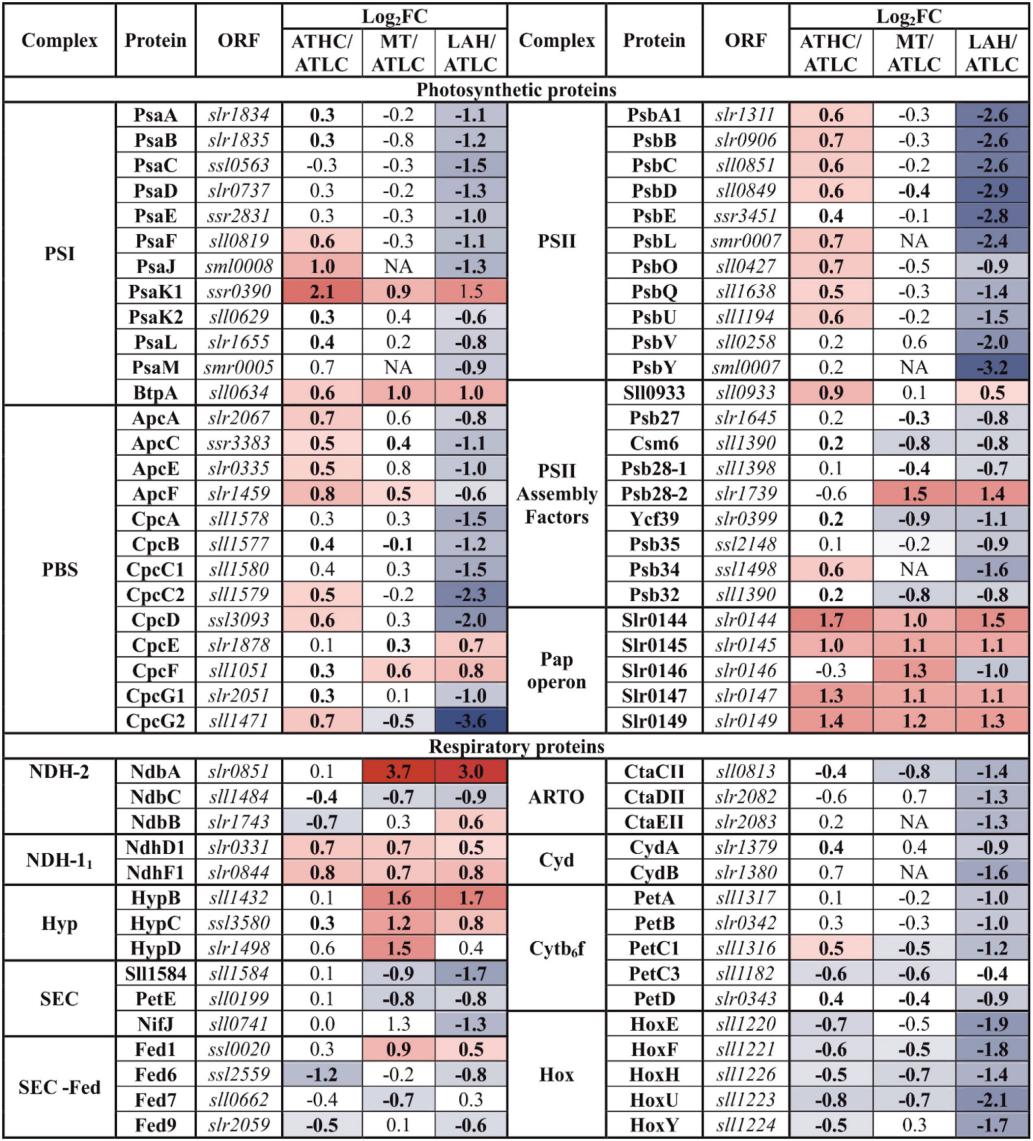

*The gradient from blue to red was applied to highlight the proteins above practical fold change threshold (−0.58 > log_2_FC > 0.58), with lowest to highest abundance, respectively, in relation to the ATLC growth condition. Statistically significant results (p <0.05) are highlighted with bold font*.

Subunits of the thylakoid cytochrome (Cyt) *b*_6_*f* complex (PetA-D), including the Rieske protein PetC1, were generally downregulated in Glc-supplemented conditions, more prominently under LAH than MT, while the periplasmic Rieske isoform (PetC3) decreased in abundance under all the carbon-rich conditions (Table 4). The NdhD1 and NdhF1 subunits of the respiratory NDH-1_1_ complex had higher abundance under ATHC, MT, and LAH than under ATLC. Cytochrome bd quinol terminal oxidases (Cyd) together with some other soluble electron carriers (SEC) decreased in abundance under the LAH condition. Under the MT condition, alternative respiratory terminal oxidase (ARTO) and ferredoxin-like protein Sll1584 (Mustila et al., 2014) were downregulated, while ferredoxin Fed1 was slightly upregulated (Table 4). The thylakoid-localized NDH-2 protein NdbA (Huokko et al., 2019) showed the highest abundance among proteins of the PSET category under the LAH and MT conditions (Table 4), while the PM-localized NdbC (Huokko et al., 2017) had lower abundance under the carbon-rich conditions (Table 4). NdbB abundance, on the other hand, was lower under ATHC but higher under the LAH condition than under ATLC.

Hydrogenase accessory proteins (Hyp) were upregulated, to various extents, under all the carbon-rich trophic conditions in comparison to ATLC, with HypB showing the second-highest upregulation in the PSET category under MT and LAH (Table 4). In comparison to the ATLC condition, HypB-D proteins were strongly induced under MT. At the same time, most protein subunits of the bidirectional hydrogenase, encoded by the *hox* operon (*hoxE, hoxF, sll1222, hoxU, hoxY*, and *hoxH*) were significantly less abundant under ATHC and MT but particularly under LAH.

Chlorophyll biosynthesis proteins ChlH, ChlD, and ChlI were at an elevated level under all the carbon-rich conditions, and the subunits of the light-independent protochlorophyllide reductase complex (ChlN, ChlL) were among the most induced proteins in comparison to the ATLC condition (Table 5). The abundance of these proteins showed a clear increase from ATHC to MT and finally to the highest level in LAH. Heme biosynthesis proteins showed differential trends under the ATHC, MT, and LAH conditions; for example, the heme oxygenase isoform (Ho1), active under aerobic conditions (Aoki et al., 2011), was upregulated under the ATHC condition and downregulated under LAH as compared to ATLC (Table 5). Cobyrinic acid, a c-diamide synthase (CobB) protein involved in the biosynthesis of other tetrapyrrole compounds, was induced under the LAH condition, while most of remaining enzymes from the pathway were not affected or did not decrease in abundance. The uroporghyrin-III C-methyltransferase (CysG) protein, a siroheme synthesizing enzyme, had high abundance under ATHC, MT, and LAH.

**Table 5:** Differential expression of proteins involved in the biosynthesis of tetrapyrrole compounds, included in the PSET category.

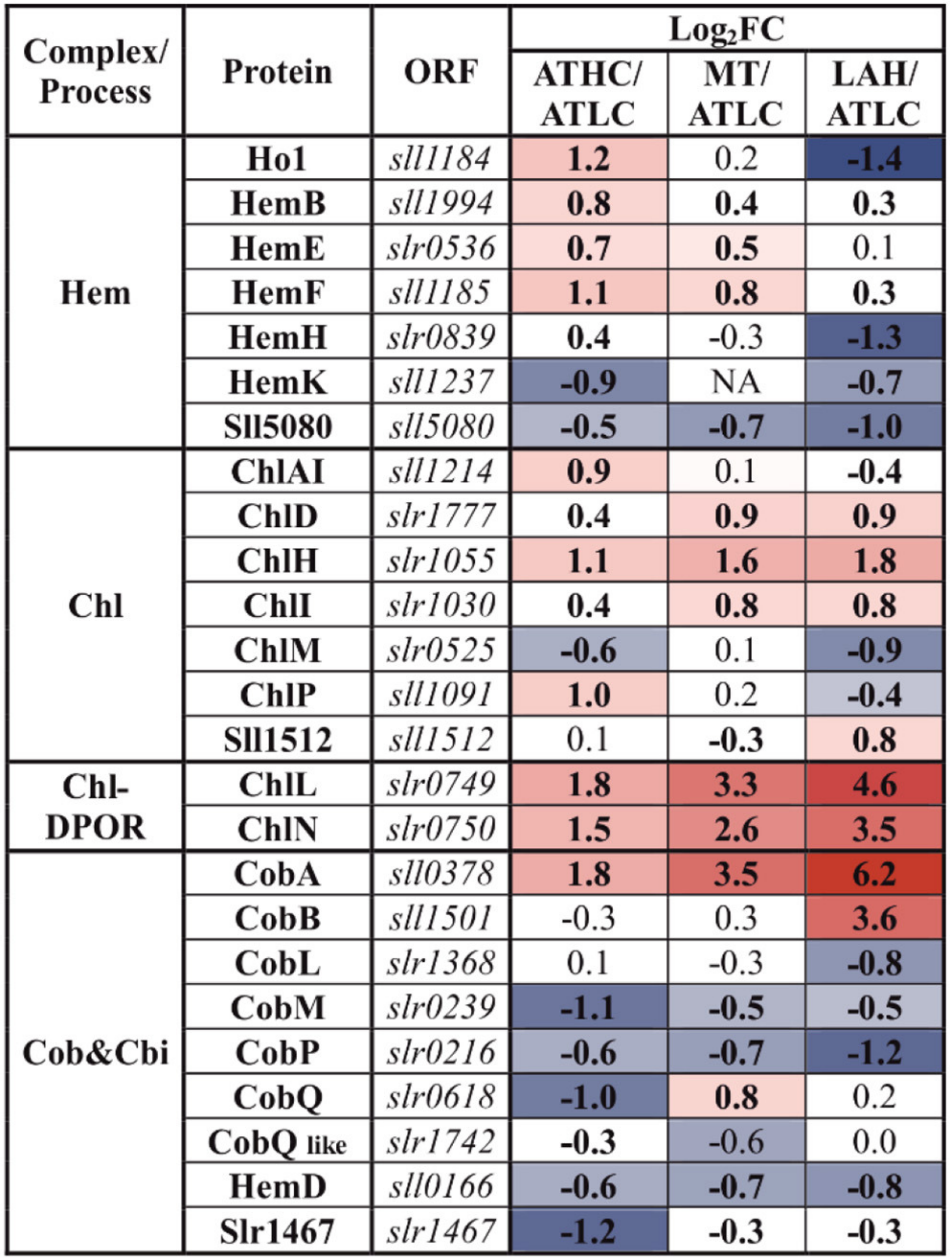

*The gradient from blue to red was applied to highlight the proteins above practical fold change threshold (−0.58 > log_2_FC > 0.58), with lowest to highest abundance, respectively, in relation to the ATLC growth condition. Statistically significant results (p <0.05) are highlighted with bold font*.

### Differential Expression of Regulatory and Maintenance Proteins (MAI) in Response to Growth Strategy

The MAI category comprises a large and very heterogeneous group of proteins involved in the maintenance and regulation of cellular functions, including a plethora of proteins with hypothetical or unknown function. Nevertheless, some MAI proteins are well-characterized, and others are gradually becoming annotated, including proteins with seminal roles in regulation of photosynthesis and cellular redox homeostasis, and protein expression for central metabolism and transport. MAI proteins with most prominent differential accumulation upon growth under different trophic conditions are shortly presented below, together with their preliminarily assigned physiological functions.

#### Regulation of Photosynthesis

Two important proteins related to non-photochemical quenching (NPQ) in cyanobacteria were among highly differentially expressed proteins depending on trophic conditions. The fluorescence recovery protein (Frp) was among the most upregulated proteins under the ATHC, MT, and LAH conditions in comparison to ATLC (Table 6). The orange carotenoid protein (Ocp) (Kirilovsky and Kerfeld, 2013) was likewise significantly upregulated under LAH compared to ATLC but was less abundant under ATHC. Alternative electron acceptors from a photosynthetic electron transfer chain (PETC) and a flavodiiron (Flv) protein (Zhang et al., 2009, 2012) were less abundant under ATHC, MT, and LAH in comparison to ATLC. The Flv2 and Flv4 proteins were identified only in ATLC samples, whereas the Flv1 and Flv3 proteins were expressed in all the growth conditions, with decreasing abundance under all the carbon-rich conditions.

**Table 6:** Differential expression of proteins from the maintenance and regulation (MAI) category with response to trophic growth condition.

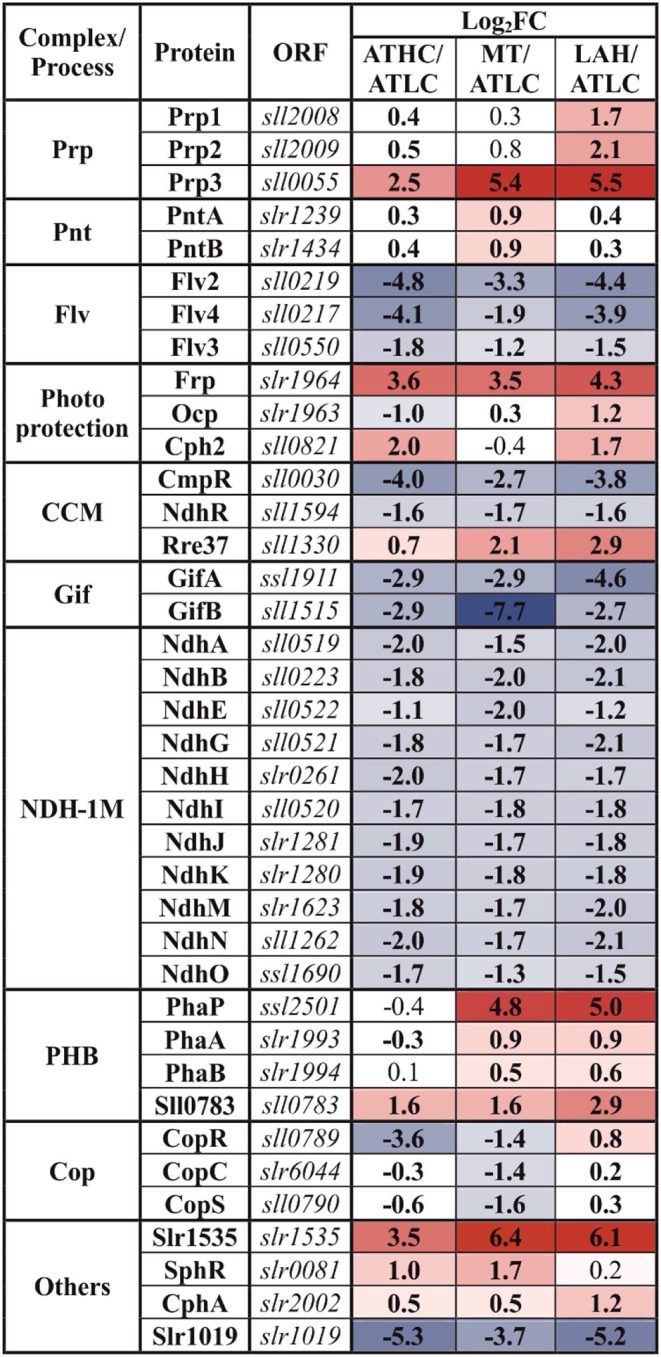

*The gradient from blue to red was applied to highlight the proteins above practical fold change threshold (−0.58 > log_2_FC > 0.58), with lowest to highest abundance, respectively, in relation to the ATLC growth condition. Statistically significant results (p <0.05) are highlighted with bold font*.

#### Redox-Related Regulation and Signaling

Trans-2,3-dihydro-3-hydroxyanthranilate isomerase (Slr1019), involved in phenazine biosynthesis, had significantly decreased abundance under all the carbon-rich growth conditions in relation to ATLC (Table 6). Phenazine is an aromatic tricyclic compound that includes two nitrogen atoms in the central ring. Its function has been associated with electron transport, cellular redox state, cell signaling, and biofilm formation (Pierson and Pierson, 2010).

#### Master Transcriptional Regulators

Regulatory proteins were assigned to the MAI category (Jahn et al., 2018) and consist of a very central group of molecules guiding the function of various cell machineries. Two main LysR type gene expression regulators of C_i_ uptake, NdhR (CcmR) (Wang et al., 2004) and CmpR (Pan et al., 2016), had reduced abundance in ATHC, MT, and LAH in comparison to ATLC (Table 6). On the contrary, the OmpR-type transcriptional regulator Rre37, described as a regulator transferring signals from light and glucose to glycolytic genes (Tabei et al., 2007), demonstrated higher abundance under all the carbon-rich conditions as compared to ATLC (Table 6). The main transcriptional regulator of nitrogen metabolism, the NtcA protein, remained constant under all the different growth modes. A two-component system CopS/CopR, regulating copper assimilation was less abundant under the ATHC and MT conditions, whereas the response regulator of phosphate uptake regulon SphR was more abundant under the ATHC and MT conditions, all in comparison to ATLC.

#### Regulators of Central Metabolism

Glutamate synthase inactivating factors GifA and GifB, highly responsive to C_i_ level, were upregulated upon CO_2_ step down under the ATLC condition (Supplementary Table 2) and maintained low abundance under all the carbon-rich conditions. The hypothetical Slr1535 and Prp3 proteins demonstrated the highest relative accumulation under the ATHC, MT, and LAH conditions (Table 6). Slr1535 contains a domain that specifies it as α-mannosidase, the protein involved in glycogen degradation (Spät et al., 2018), while Prp3 has been characterized as a processing metalo-peptidase with unknown target(s) (Sokolenko et al., 2002). Two other processing metalo-peptidases, Prp1 and Prp2, involved in degradation of GifA (Galmozzi et al., 2007) accumulated under the ATHC and LAH condition (Table 6). However, a hypothetical protein, Ssl2501, is among the most induced proteins under the MT and LAH conditions, and has been characterized as phasin (PhaP), a protein controlling the amount and size of polyhydroxybutyrate (PHB) granules (Hauf et al., 2015). Other proteins involved in PHB synthesis, such as acetyl-CoA acetyltransferase PhaA, acetoacetyl-CoA reductase PhaB, and Sll0783 (Schlebusch and Forchhammer, 2010; Hauf et al., 2015), likewise accumulated in LAH and MT (Table 6). Elevated abundance under MT and, to a lesser extent, under ATHC and LAH in comparison to ATLC was recorded for pyridine nucleotide transhydrogenase PntAB, responsible for adjusting NADH:NADPH ratio (Kämäräinen et al., 2017) (Table 6).

The common core unit NDH-1M, composed of the NdhA, B, E, G-K, and M-O subunits and present in all NDH-1_1−4_ complexes, was qualified to the MAI category in our analyses, since the NDH-1_1−2_ and NDH-1_3−4_ complexes maintain different functions in cyanobacterial cells. All common subunits had lower abundance under the three carbon-rich conditions (ATHC, MT, and LAH) compared to ATLC (Table 6).

## Discussion

### Trophic Conditions Govern Growth Rates and Proteome Profiles

Aquatic photosynthetic microorganisms modulate their growth mode among autotrophy, mixotrophy, and heterotrophy by tuning the metabolic, bioenergetic, and transport pathways according to the availability of nutrients, particularly carbon, and light. However, knowledge gaps regarding pathway preferences or discrimination in different trophic conditions are numerous. This study focused on identifying metabolic pathway modulations that govern the growth and photosynthesis of *Synechocystis* cells under autotrophy at low (ATLC) or high CO_2_ (ATHC), as well as under Glc-supplemented mixotrophy (MT) and light-activated heterotrophy (LAH) at low CO_2_. Identical continuous irradiance conditions (50 μmol photons m^−2^ s^−1^) were applied for the ATLC, ATHC, and MT strategies, while LAH occurred in darkness with only 10-min illumination every 24 h. With respect to the general availability of carbon, the ATHC, MT, and LAH trophic conditions were collectively denoted “carbon-rich” irrespective of organic or inorganic origin, whereas ATLC represented the natural low-carbon reference condition.

**Figure 5:**
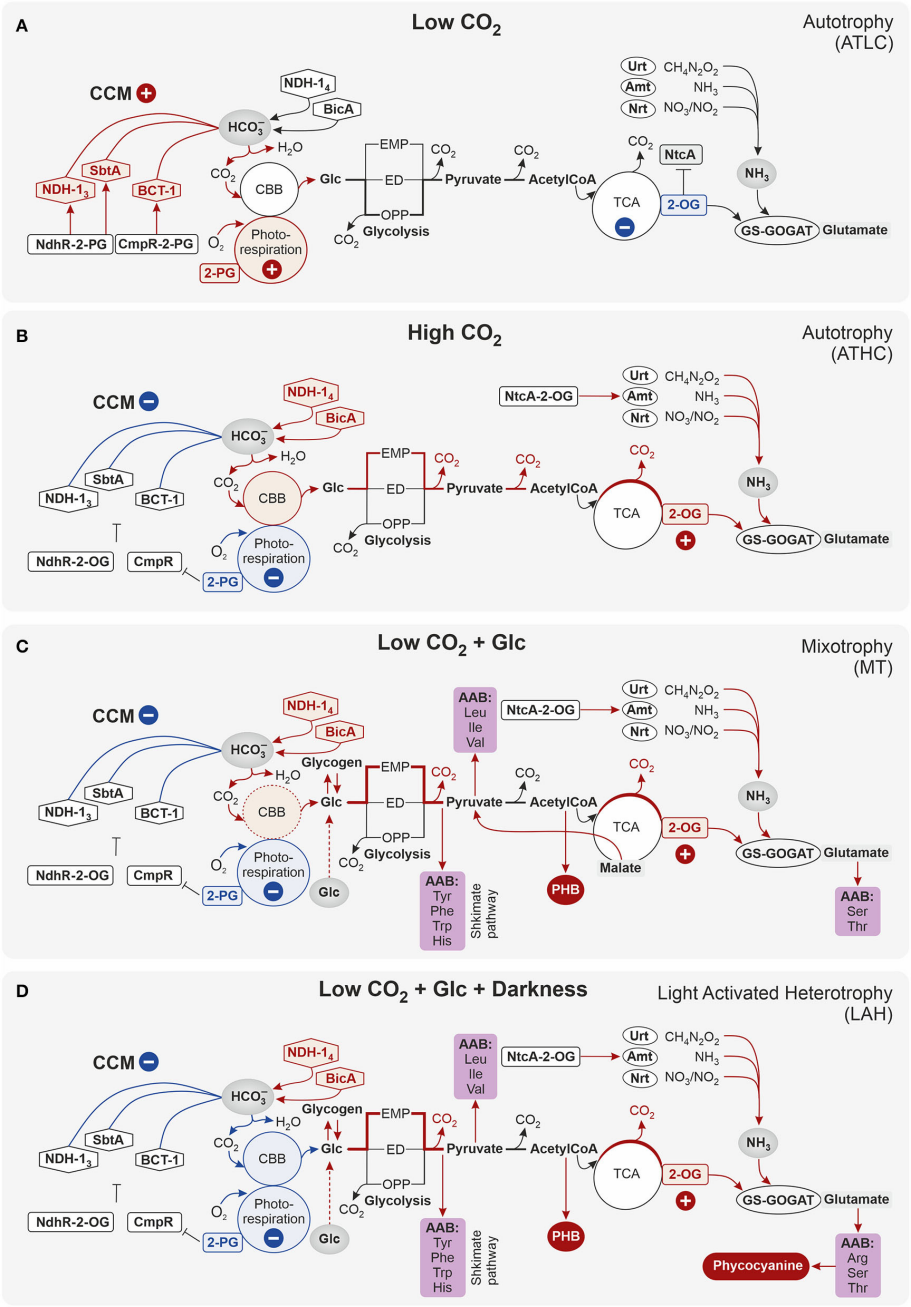
Schematic representation of the regulation of carbon metabolism in Synechocystis sp. PCC 6803 cells grown under **(A)** low-carbon autotrophy (ATLC) and **(B–D)** carbon-rich ATHC **(B)**, MT **(C)**, LAH **(D)** conditions. The main sensors of carbon availability in the cells are 2-phosphoglycolate (2-PG), the product of photorespiration, and 2-oxoglutarate (2-OG), an intermediary product in TCA cycle. In low CO_2_ autotrophy (ATLC), 2-PG is typically at high level together with CCM (red symbol), while 2-OG-regulated nitrogen assimilation is downregulated (blue symbol). Differently from (ATLC), under carbon-rich conditions, independently on the length of illumination or the source of carbon, the prevalence of 2-OG with respect to 2-PG causes downregulation of CCM (blue arrow), strengthens RuBisCo carboxylase activity (red color) and induces nitrogen transport (red arrow) that is immediately build in the 2-OG carbon skeleton to provide glutamate. The NAD(P)H/NADP^+^ ratio in the cell is maintained by different processes depending on carbon availability.

**Figure 6:**
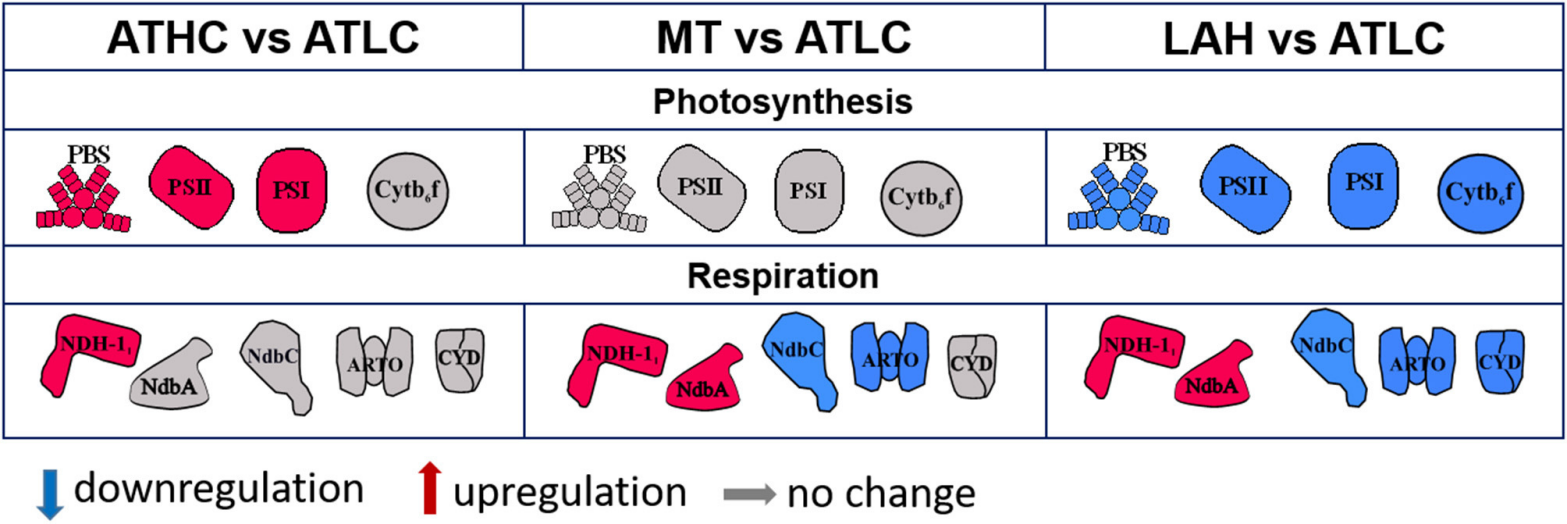
Summary of differentially regulated complexes or proteins from photosynthesis and respiration processes under the ATHC, MT, and LAH conditions compared to the reference ATLC condition. Red color, increase (log_2_FC > 0.58); gray color, no change (−0.58 < log_2_FC <0.58); blue, decrease (−0.58 > log_2_FC) in abundance with *p* < 0.05.

The experimental cultures exhibited different growth rates that corelated with carbon and light availability. ATHC and MT demonstrated enhanced growth due to a surplus of CO_2_ and Glc, respectively (Figure 1). The slow growth of the ATLC and LAH cultures was due to limited CO_2_ and light, respectively. Unique metabolic strategies adapted in the four growth conditions were reflected in differences in proteome mass fraction distribution (Figures 3, 4). These results highlighted especially how carbon metabolism and energy production processes are adjusted to the needs of diverse trophic conditions (discussed below). More detailed information about the metabolic pathways employed under specific trophic conditions was obtained from assessing the DE of single proteins and subunits of protein complexes in the three carbon-rich growth modes with ATLC as a reference condition (Tables 2–6). These proteome-based observations allowed insights into physiological processes that gained or lost importance under each trophic growth strategy, as summarized in Figures 5, 6.

### Acquisition of Carbon/Nitrogen Balance

Sensing Carbon/Nitrogen (C/N) balance by the accumulation of 2-OG or 2-PG is an important requirement for adjusting metabolism and nutrient uptake to maintain metabolic homeostasis in cyanobacteria (for a review, see Forchhammer and Selim, 2019). The proteome of each carbon-rich trophic strategy revealed an accumulation of proteins involved in N acquisition and metabolism, including nitrate, ammonia, and urea transporters and nitrate reductases, at significantly higher levels compared to low-carbon ATLC (Table 2). Such high levels of N import and metabolism imply high levels of cellular 2-OG in all the carbon-rich conditions, independently of whether the carbon source was Glc or surplus CO_2_ (Figure 5). Low-carbon conditions, on the other hand, lead to low 2-OG levels (Burnap et al., 2015), evidenced here by the low expression of proteins involved in N-assimilation-related processes (Figure 5). Nonetheless, the C/N balance sensing proteins P_II_ and NtcA (Forchhammer and De Marsac, 1994; Tanigawa et al., 2002; Vázquez-Bermúdez et al., 2002; Watzer et al., 2019) showed stable abundance levels among all the four growth conditions under study, providing evidence that their regulatory activity is not necessarily evidenced by the DDA data (Supplementary Table 1) but is rather modulated by sensing allosteric effectors 2-OG, ATP/ADP, NAGK, and PipX (Forcada-Nadal et al., 2018) or/and by post-translational modifications (PTM) (Lee et al., 2000; Watzer et al., 2019).

The carbon metabolism of cyanobacteria is highly dependent on the availability of CO_2_ for carbon assimilation by the Calvin–Benson–Bassham (CBB) cycle, with CCMs playing an integral role in the capacity of cyanobacteria to thrive under low carbon conditionsby allowing for the enhancement of the carboxylation activity of RuBisCO and decreasing photorespiration (Burnap et al., 2015; Kaplan, 2017) (Figure 5). ATLC uniquely displayed nearly twice the mass fraction in the CCM subproteome (Figure 4B), particularly in its inducible components (BCT1, NDH-1_3_, and StbA/B) (Table 2). Under these conditions, the enhanced binding of the photorespiration product (2-phosphoglycolate, 2-PG) by the two regulatory proteins, CmpR and NdhR, enables the transcription of CCM genes (Jiang et al., 2017) and, indeed, the accumulation of their encoded proteins was evident from our data sets (Table 2). CmpR-2-PG likely induced the transcription of genes encoding inducible HCO${}_{3}^{-}$ transporter BCT1, while NdhR-2-PG upregulated the expression of the *sbtA/B* and *ndh1*_3_ operons and the *ndhR* gene itself (reviewed in Burnap et al., 2015) (Figure 5).

Under high carbon conditions, on the other hand, the accumulation NdhR-2-OG suppressor complex is known to reduce the abundance of inducible CCM proteins and NdhR itself (Jiang et al., 2017), which was clearly evident in all the carbon-rich conditions in our study (Tables 2, 6). These findings are in line with the low abundance of inducible CCM proteins together with the downregulation of glycolate metabolism components (Table 2), thus indicative of low level of photorespiration in the presence of Glc as well as under high CO_2_ (Burnap et al., 2015). Meanwhile, the constitutively expressed low affinity C_i_ transport systems (BicA and NDH-1_4_) provided the means for C_i_ uptake under all the carbon-rich conditions (Table 2; Figure 5). In addition, the downregulation of both CBB cycle proteins and carboxysome-building proteins under these conditions was likely operated *via* the RbcR (Ycf30) activator of CBB genes (Carmel, 2011; Tomar et al., 2017), being slightly downregulated under all the carbon-rich conditions in comparison to ATLC (Table 2).

### Production of Reducing Power and ATP *via* Metabolic Processes

Reducing power (NAD(P)H) and chemical energy (ATP), both necessary to run metabolic processes, are derived prevalently from photosynthesis under autotrophy and from glycolysis and respiration in darkness or upon heterotrophic growth (Figure 5). It has been hypothesized that the presence of three glycolytic routes in *Synechocystis*, the oxidative pentose phosphate (OPP), Entner-Doudoroff (ED), and Embden-Meyerhof-Parnas (EMP) pathways, represents different opportunities for quick acclimation to dynamic changes in nutrients and/or light availability (Chen et al., 2016; Wan et al., 2017; Makowka et al., 2020; Lucius et al., 2021). The OPP pathway has been demonstrated as the main glycolytic route and the source of NADPH in darkness but maintains only residual activity under autotrophy (Welkie et al., 2019; Makowka et al., 2020). In this study, the abundance of OPP pathway proteins was hardly affected by the trophic condition, irrespective of carbon supply or availability of light (Table 3). Conversely, the increased abundance of phosphofructokinase (Pfk1) and phosphoglycerate mutase (Pgm) under LAH and MT suggests enhanced flow through the EMP pathway in the presence of Glc. Notably, the essential regulator of glycolytic genes, Rre37 (Tabei et al., 2007; Azuma et al., 2011), was found upregulated under all the carbon-rich conditions in this study (Table 6). Despite the observed changes in abundance of some glycolytic enzymes, it remains unclear whether the measured abundance of the protein corresponds with its activity, as many glycolytic enzymes are allosterically activated (Feng et al., 2014; Nishiguchi et al., 2019, 2020). Thus, the activity of a particular glycolytic pathway is difficult to predict from the proteome data without additional protein structure analyses or metabolite screening (Feng et al., 2014). The glycolytic routes generate reduced power in the form of NADH or NADPH. The balance between these two forms is maintained by a cytoplasmic pyridine nucleotide transhydrogenase, PntAB, which, in *Synechocystis*, is specifically directed toward NADPH (Kämäräinen et al., 2017). The increased abundance of PntAB under the MT condition (Table 6) implies the predominance of NADH over NADPH, possibly due to enhanced carbon flux through the EMP pathway, as compared to fully autotrophic growth mode. Some increase in PntAB abundance was also observed under ATHC and LAH due to a greater need for NADPH for CO_2_ fixation and the lack of photosynthetic NADPH production, respectively.

### Modulation of Photosynthetic and Respiratory Electron Transfer in Response to the Trophic Growth Strategy

The main bioenergetic hubs in cyanobacteria comprise the mixed respiratory and photosynthetic electron transfer pathways in the TM and the separate respiratory pathway(s) occurring in the PM (Mullineaux, 2014; Liberton et al., 2016; Baers et al., 2019; Liu and Zhang, 2019; Rast et al., 2019). As for respiratory complexes in the PM, no distinct differences were evident between the autotrophic conditions (ATLC and ATHC), while MT resulted in lower abundance of PM-located respiratory complexes (NdbC, ARTO, and Cyd) and even fewer were present in the LAH condition (Figure 6; Table 4). The common and specific feature in both Glc-supplemented conditions, MT and LAH, was distinct accumulation of the type 2 NAD(P)H dehydrogenase (NDH-2) protein NdbA (Table 4), which locates in the TM (Huokko et al., 2019) and potentially mediates electrons from NAD(P)H to the PQ pool (Howitt et al., 1999). Thus, the PM-located respiratory activity appears not to be of particular importance for growth under an organic carbon source. Instead, ATP seems to be primarily sourced from thylakoids, where NdbA is likely to act as an electron carrier from Glc degradation to the thylakoid electron transfer chain beside its other potential functions (Desplats et al., 2009; Saroussi et al., 2016). In addition, the respiratory NDH-1_1_ complex accumulated in the TM, but this was not specific only for MT and LAH but also occurred under ATHC and coincided with downregulation of Flv1/3 proteins (Table 6). It, therefore, appears that in all the carbon-rich conditions, the availability of NADPH is not a critical factor, and that instead the cells invest in efficient ATP production *via* NDH-1 cyclic electron transfer. Flexibility between cyclic NDH-1_1_ and Flv1/3 electron transfer routes in *Synechocystis* cells has been assigned also for the protection of PETC against over reduction (Nikkanen et al., 2020).

Distinct upregulation of PETC complexes takes place upon transfer of *Synechocystis* from LC to HC conditions to meet the high ATP and NADPH demands to support efficient CO_2_ fixation and rapid growth under ATHC (Battchikova et al., 2010; Muramatsu and Hihara, 2012). This can also be deduced from the proteome mass fraction distribution in the PSET subcategory (Figure 4A). Under MT, the distribution of the proteome mass fraction for photosynthetic complexes in PSET was very similar to that in ATHC, demonstrating the importance of photosynthetic electron transport in supplement to glycolysis under mixotrophy. The extent of the role of photosynthesis in MT might be dependent on the growth state of cells and strains under examination, as attenuation of photosynthesis in *Synechocystis* grown under mixotrophy has also been reported (Solymosi et al., 2020). LAH cells, on the contrary, were severely depleted in thylakoid light-harvesting and photosynthetic electron transfer complexes PSII, PSI, and Cyt*b*_6_*f* in comparison to other trophic conditions (Figures 3, 4A and Table 4). It has been shown earlier that PSII is not functional under the LAH condition (Anderson and McIntosh, 1991; Huokko et al., 2019), while PSI still remains partially functional (Barthel et al., 2013; Huokko et al., 2019). Unlike in a previous study (Plohnke et al., 2015), downregulation of all photosynthetic subunits under LAH occurred in this study (Figures 3, 4 and Table 4), indicating a low level of photosynthetic and respiratory electron transfer in the TM and reiterating strong reliance on glycogen catabolism (Table 2) to sustain growth. However, the induction of iron uptake as well as iron-sulfur cluster protein biosynthesis under both glucose-supplemented conditions indicates the need for electron carriers and important cofactors to ensure redox homeostasis under LAH and MT.

Despite a general trend for downregulation of photosynthesis subunits in LAH relative to ATLC, several proteins associated with photosystems were upregulated (Table 4), including Psb28-2 (Sakata et al., 2013; Bečková et al., 2017) and the *pap* operon involved in PSII biogenesis (Wegener et al., 2008; Rast et al., 2016), as well as the PSI assembly protein BtpA (Bartsevich and Pakrasi, 1997; Zak et al., 1999). Additionally, the phycocyanobilin lyases CpcE and CpcF that are responsible for the attachment of bilin chromophores to apoproteins (Scheer and Zhao, 2008; Zhao et al., 2017; Kronfel et al., 2019) were induced relative to ATLC (Table 4). Although these proteins were also upregulated under the other trophic conditions, in comparison to ATLC, their abundance in LAH was unexpected and suggested that rapid activation of photosynthetic complexes is important to capitalize on brief daily exposure to light. Furthermore, both light-dependent and light-independent chlorophyll synthesis (LDCS and LICS respectively) pathways were induced under all the carbon-rich conditions (Table 5); however, LICS showed the highest increase under LAH in line with previous observations (Fang et al., 2017). It is, therefore, conceivable that the induction of two *pap* operon proteins Slr0144 and Slr0147 (Table 4) with a chlorophyll-binding domain in their structures (Wegener et al., 2008) transiently neutralizes the disadvantage because of free pigments under LAH where LICS proteins accumulate (Table 5). In addition, upregulation under LAH of two putative ferredoxins (containing Fe-S clusters) of the *pap* operon, Slr0148, and Slr0150, possibly reflects enhanced activity of LAH-specific metabolic pathways requiring these cofactors. Finally, the abundance of the Slr1051 tetratricopeptide repeat protein (TRP), previously shown to be involved in PSII repair under high light conditions (Yang et al., 2014), increased in abundance under LAH (Table 2). Overall, the *pap* operon proteins were strongly elevated under all the carbon-rich conditions (Table 4), implying their importance although still poorly understood, under the conditions of enhanced carbohydrate metabolism.

Conversely, the abundance of photoprotective Flv proteins, particularly Flv2, 3, and 4, was strongly diminished under all the carbon-rich conditions in comparison to the ATLC reference condition (Table 6), reflecting a balanced state of the photosynthetic apparatus that appears to be related to the availability of carbon (Zhang et al., 2009, 2012; Battchikova et al., 2010). The OCP protein was likewise downregulated under ATHC (Table 6) but upregulated under the LAH condition, where it may have a role in quenching excess light energy in sudden and short daily illumination periods. On the other hand, the ability of OCP to bind a chromophore may consist of a way to neutralize the potentially harmful activity of free carotenoids under the LAH condition (Melnicki et al., 2016). The strong upregulation of the OCP-inactivating protein FRP (Boulay et al., 2010) in all the carbon-rich conditions, reported also in previous proteomic studies (Kurian et al., 2006; Plohnke et al., 2015), may indicate an additional but still unknown function for FRP.

### Glucose Supplementation Upregulates the Proteome for Amino Acid and Protein Synthesis

A clearly higher accumulation of protein mass in the RIB (ribosome and protein production) category was the most distinct proteome feature shared by the Glc supplemented MT- and LAH- grown *Synechocystis* cells in comparison to both AT conditions (Figure 3). The TCA cycle provides building blocks for biosynthesis of amino acids, the substrate for protein translational machinery. The presence of multiple branches from the TCA cycle, such as the GABA shunt, glyoxylate shunt, and OgdA/SsaD bypass, provides flexibility for cyanobacterial physiology and consequently allows for the acclimation of cells to alterations in nutrient availability (Zhang et al., 2016). The Glc-supplemented conditions of MT and LAH slightly enhanced the accumulation of Icd (*slr1289*) that catalyzes the oxidative step leading to 2-OG formation, although all other components of the TCA cycle were moderately downregulated in all the carbon-rich conditions compared to ATLC (Table 2). The induced flux toward 2-OG coincided with the increased efficiency of the N fixation process evidenced by the high abundance of GlnN and low contents of GifA and GifB under all the carbon-rich conditions, which also correlates with the increased N uptake discussed earlier. Furthermore, the strong downregulation in Glc-supplemented conditions and, to a lesser extent, in ATHC of enzymes, such as succinate dehydrogenase (SdhA, SdhB), succinyl-CoA synthetase (SucC and SucD), and FumC, catalyzing the consecutive reductive steps of the TCA cycle, strongly implied the redirection of carbon flow toward amino acid biosynthesis. Under the MT condition, enhanced flow through the malic shunt was evidenced by increased abundance of Me, which leads to an alternative way of pyruvate biosynthesis (Table 2, Figure 5) (You et al., 2015).

Under the LAH and MT conditions, the induced flow *via* GS-GOGATis likely directed into ornithine and AAB, as deduced from the strong accumulation of ArgB and ArgD enzymes (Table 2). It is conceivable that in Glc supplemented conditions, glutamate is incorporated in serine, threonine, and arginine. ArgB (NAGK) interacts with the P_II_ protein and controls arginine (Arg) synthesis *via* feedback inhibition determining the limiting step in Arg biosynthesis (Forchhammer and Selim, 2019), while ArgD is a subsequent enzyme catalyzing the reaction of N-acetylornithine biosynthesis (Xiong et al., 2014). In addition, enhanced flux toward pyruvate under Glc-supplemented conditions results in enhanced aliphatic and aromatic AA biosynthesis (Figure 5), evidenced by the accumulation of AroK/AroH, HisC/HisD, and TrpC (Table 2). Furthermore, the upregulated Sll0934 protein, predicted to encode a 3-deoxy-7-phosphoheptulonate synthase (AROA2 or DAHP) (Ogawa et al., 1994), which catalyzes one of the steps in the shikimate pathway biosynthesis (Brey et al., 2020), corroborates the enhancement of aromatic AAB.

### Differential Accumulation of Pathway Enzymes for Storage Polymers Reveals Unique Strategies in Different Trophic Modes

Glycogen synthesis and degradation cycle are an important energy buffer and are recently shown to be also crucial for photosynthesis activation (Cano et al., 2018; Shinde et al., 2020). Enzymes involved in glycogen synthesis did not show differential accumulation in this study, while several glycogen catabolism enzymes diminished in abundance under Glc supplementation in MT and LAH (Table 2). The higher abundance of the GlgP2 isoform, as well as Pfk2 in MT and LAH, correlates with the induction of the biosynthesis of PHB, another carbon storage polymer that accumulates in *Synechocystis* under N or phosphate deprivation and functions as an important electron sink and source of building blocks for AAB (Panda et al., 2006; Panda and Mallick, 2007; Schlebusch and Forchhammer, 2010; Koch et al., 2019, 2020).

A striking difference in the accumulation of phosphate transporters (Psts) was observed for the two *Synechocystis* cultures supplemented with Glc, being strongly upregulated in MT and, conversely, downregulated in LAH, compared to the autotrophic conditions (ATLC and ATHC, Table 2). Induction of polyphosphate kinase Ppk together with Psts transporters in MT indicates enhanced synthesis of polyphosphates (polyPs) composed of orthophosphate residues linked with high-energy phosphoanhydride bonds (Gómez-García et al., 2003; Gomez-Garcia et al., 2013). Accumulation of polyPs granules in *Synechocystis* cells and algae grown under mixotrophy has been previously shown (Plohnke et al., 2015; Wu et al., 2021) and reflects the high cellular energy level in the MT condition due to the presence of simultaneously induced sugar catabolism and photosynthesis. Neutralization of anionic polyPs with positive charges could explain the high abundance of potassium ion channels (Kdps) observed under the MT condition (Table 2). Maintenance of phosphate storage in *Synechocystis* is considered as an adaptation to changes occurring in environmental conditions and energy reservoirs (Voronkov and Sinetova, 2019; Sanz-Luque et al., 2020).

The upregulation of N uptake and metabolism, including biosynthesis of N-rich AA like arginine in the LAH condition, coincided with the upregulation of cyanophycin synthetase CphA (Table 6). CphA is the main enzyme in the biosynthesis of cyanophycin, a polymer composed of equimolar aspartate and arginine residues, and forms granule peptide structures visible in cells grown under LAH conditions (Plohnke et al., 2015). Cyanophycin accumulates in unbalanced growth or under excess nitrogen, plays an important role in cell adaptation to different nutritional conditions, and buffers C and N availability (Watzer and Forchhammer, 2018).

### Autotrophic and Heterotrophic Proteomes Are Balanced Under Mixotrophic Growth

Taken together, it is apparent that the *Synechocystis* cells benefited from both the autotrophic and heterotrophic traits in unique combinations during MT growth. As the differences between the trophic growth strategies were dependent on the quantity as well as the quality of the carbon source and on the availability of light for photosynthesis, the proteomes of *Synechocystis* cells harvested for investigation at similar OD_750_, mostly reflected the differences in carbon acquisition and bioenergetic (photosynthesis and respiration) systems as well as in protein and amino acid biosynthesis, and accumulation of transporters and polymer storage.

The PSET proteome category, comprising the two photosystems, Cyt*b*_6_*f* , ATPase, NDH-1_1_, and NDH-2, soluble electron carriers and ARTO, was present in surprisingly similar abundance in the total proteomes of *Synechocystis* from both autotrophic and the MT conditions. On the other hand, these proteomes distinctively but differentially diverged from the Glc-supplemented LAH proteome by a significant decrease in the accumulation of PSI and PSII as well as by increase in ATP synthase protein mass fraction (Figure 4A).

A common feature of ATHC, MT, and LAH was a shutdown of the energy-consuming inducible CCM (SbtA/B, BCT1, and NDH-1_3_) that comprises a considerable proteome mass fraction in ATLC (Figures 3, 4B). It is conceivable that the levels of accumulated 2-OG and 2-PG downregulated the inducible CCM to balance the cellular C/N ratio independently of the inorganic or organic source of carbon. It is likely that high C/N under all the carbon-rich conditions led to an enhanced flux of carbon through the GS-GOGAT pathway, which in turn enhanced N uptake and metabolism, in comparison to ATLC. Under the LAH and MT conditions, the surplus of carbon was derived from glycolysis and was incorporated into arginine and AAB, as well as converted to PHB.

The proteome data revealed strong upregulation of another respiratory NDH-2 protein, NdbA, together with the respiratory NDH-1_1_ complex localized in the TMin MT and LAH strategies, and downregulation of respiratory pathways located in the PM, particularly the NDH-2 protein NdbC and ARTO, in comparison to ATHC and ATLC.

These results suggest that the respiratory electron transfer complexes in both TM and PM provide ATP for enhanced carbon fixation in the ATHC condition as compared to ATLC. Instead, respiration upon MT mode seems to primarily rely on high abundance of NdbA and the NDH-1_1_ complex in the TM, while at the same time, PM respiratory components diminished in comparison to ATLC. It is also worth noting that the downregulation of auxiliary electron transfer pathways catalyzed by Flv1-4, as well as other photoprotective proteins, occurred similarly in MT and ATHC. Furthermore, the scarcity of photorespiratory proteins provides evidence that RuBisCO primarily functions in CO_2_ fixation under MT conditions rather than in the oxygenation reaction that leads to photorespiration and loss of fixed carbon, thus mimicking in this respect the autotrophic ATHC condition.

In LAH, with only 10 min of daily illumination, depletion of respiratory complexes from the PM as well as the photosynthetic and PBS proteins from the TM was evident when compared to all the other trophic conditions. However, the high level of the ATP synthase and the induction of thylakoid-localized NDH-2 (NdbA) and NDH-1_1_ complexes suggest rigorous respiration and ATP synthesis primarily in the TM. Although LAH thylakoids mainly support dark respiration, they also host partially functional PSI centers (Huokko et al., 2019) that are essential during a short but obligatory diurnal illumination period that is required for *Synechocystis* growth under LAH by mechanism(s) that remains elusive.

It is concluded that upon flexible mixotrophic growth under low CO_2_ and supplemental Glc (MT), *Synechocystis* relies on the use of a plethora of different physiological traits that also partially develop upon ATHC. These include downregulation of the CCM and enhanced N-metabolism. An additional feature of MT proteome, missing from all the other trophic condition proteomes, including ATHC, is profound accumulation of Psts as well as induction of the malic shunt of the TCA cycle (Table 2 and Figure 5). The latter feature likely provides an invaluable asset for mixotrophic cultures to enhance their metabolism upon the availability of organic carbon. Surplus of produced energy when both autotrophic and heterotrophic growth strategies coexist is stored in polyPs granules neutralized with potassium ions. Their likely role for cyanobacteria in natural environments of low ambient CO_2_ is to provide trophic flexibility in highly dynamic environments, for example, to cope with nutrient exhaustion following phytoplankton blooms. From a biotechnology viewpoint, it is clear that metabolic pathways show great plasticity according to the trophic growth mode and could be utilized as an asset for efficient production of target molecules like aromatic amino acids as an example.

## Data Availability Statement

The mass spectrometry proteomics data generated and analyzed for this study have been deposited to the ProteomeXchange Consortium *via* the PRIDE partner repository with the dataset identifier PXD030630 (http://www.ebi.ac.uk/pride/archive/projects/PXD030630).

## Author Contributions

DM-P, TH, YA, and E-MA made the experimental design. DM-P and TH conducted the experiments. DM-P, SK, and E-MA analyzed the proteomics data. DM-P, PG, and E-MA interpreted the data and wrote the manuscript. All authors contributed to the revision of the manuscript. All authors contributed to the article and approved the submitted version.

## Funding

The authors acknowledge the Jane and Aatos Erkko Foundation, Turku Collegium for Science and Medicine (TCSM), and NordForskNCoE, NordAqua (Project 82845).

## Conflict of Interest

The authors declare that the research was conducted in the absence of any commercial or financial relationships that could be construed as a potential conflict of interest. The handling editor LL declared a past collaboration with the author TH.

## Publisher's Note

All claims expressed in this article are solely those of the authors and do not necessarily represent those of their affiliated organizations, or those of the publisher, the editors and the reviewers. Any product that may be evaluated in this article, or claim that may be made by its manufacturer, is not guaranteed or endorsed by the publisher.
